# A case of twin reversed arterial perfusion (TRAP) sequence managed conservatively

**DOI:** 10.11604/pamj.2019.32.54.15480

**Published:** 2019-01-30

**Authors:** Enesia Mudenha Ziki, Zvavahera Mike Chirenje, Mugove Gerald Madziyire

**Affiliations:** 1 Department of Obstetrics and Gynaecology, University of Zimbabwe, College of Health Sciences, Harare, Zimbabwe

**Keywords:** TRAP, pump twin, doppler

## Abstract

The TRAP sequence, also known as acardiac twinning is a rare complication that is unique to monochorioinic multiple pregnancies affecting 1% of monochorioinic pregnancies and about 1 in 35000 of all pregnancies. In TRAP, blood flows from the umbilical artery of the pump twin to the umbilical artery of the perfused twin through artery to artery (AA) anastomosis. The perfused twin has poor development of the upper extremities and the normal or pump twin is at risk of a poor perinatal outcome. This is a report of a patient with TRAP sequence diagnosed in the second trimester who was managed conservatively and had a good outcome for the normal twin.

## Introduction

The TRAP sequence is a rare complication unique to monochorioinic multiple pregnancies affecting 1% of monochorioinic pregnancies and 1 in 35000 of all pregnancies [1]. These figures are used widely in literature yet they are based on data dating back to 1953 but the incidence appears to be higher now because of use of first trimester ultrasound scan and because of assisted reproductive techniques. The incidence has been quoted as 2.6% of monochorioinic twins and between 1 in 9500 and 11000 pregnancies in a 2015 study [2]. In the TRAP sequence the normal twin pumps blood to the perfused twin through the umbilical arteries via the AA anastomosis creating a reversed circulation in this twin. This is possible because there is no functional heart in the acardiac twin which would normally provide forward flow and high systemic pressure. The anomalous twin receives poorly oxygenated blood and this is extracted by the lower part of the body, resulting in further oxygen depleted blood reaching the upper part of the body and eventually in varying degrees of abnormal development of the head, heart and upper limbs [3]. The normal twin is at a risk of high output failure, preterm birth and poor perinatal outcome. The pump twin usually has no congenital malformations but has an increased mortality rate between 35 and 50% [4, 5]. The abnormal twin may appear as a heterogeneous mass on ultrasound scan or as intrauterine foetal demise as in the case presented.

## Patient and observation

**Figure 1:**
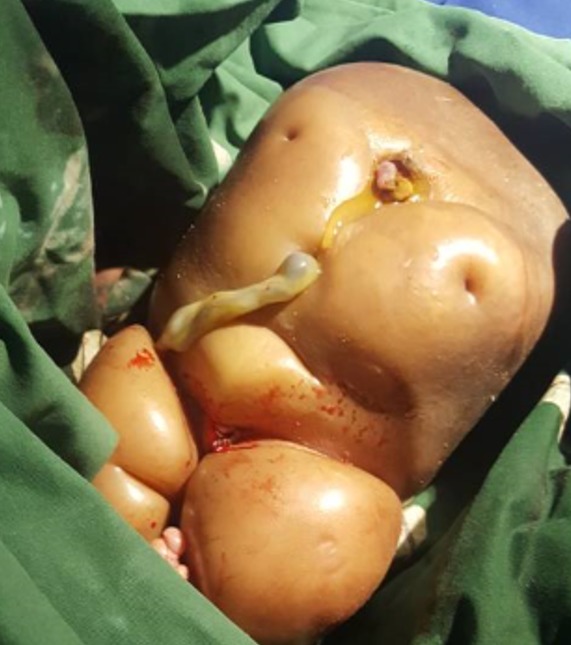
Acardiac twin weighing 3000g. Acardius acephalus, the most common degree of abnormal development

Mrs. CD, a 27 year old Para 2 gravida 3 patient was referred from a district hospital with a diagnosis of multiple pregnancy with intrauterine demise of a co-twin at 26 weeks gestation. She had no complaints at presentation and had not experienced excessive symptoms of pregnancy earlier on in the pregnancy. She believed her abdomen was growing at a normal rate and she had felt quickening a few weeks before. The pregnancy was planned and she booked the pregnancy at the district hospital a week prior to presentation. She had normal vaginal deliveries of her first 2 children with no antepartum or intrapartum complications but was diagnosed with chronic hypertension during her 6 week post-natal visit after delivery of her second child. She was started on enalapril and nifedipine but she defaulted treatment 2 months before falling pregnant. Her method of contraception was a combined oral contraceptive (Lofeminal) which she stopped taking one month before she fell pregnant. Nifedipine and methyldopa were commenced at 26 weeks gestation when the blood pressure was noted to be elevated and the blood pressure was well controlled thereafter. Clinical examination was unremarkable. An ultrasound scan showed monochorioinic diamniotic twins with normal liquor volume in the viable twin but there was gross oedema of the foetal skin and no skull bones in the non-viable twin. Later scans suggested that the foetal parts were encapsulated in a cystic mass suggestive of a partial mole. Umbilical artery doppler studies showed similar indices for both foetuses and there was no mention of reversed flow in the scans at 26 weeks. A plan to manage her expectantly was made and she was followed up by two weekly ultrasound scan and doppler velocimetry. The patient was counselled on the risks for preterm birth, cardiac failure and higher perinatal mortality associated with the condition. She was counselled for an elective caesarean section (CS) once the foetus reached viability. Follow up scans and doppler velocimetry showed that growth of the pump twin was below the 5^th^ percentile but the weight of the acardiac twin was difficult to estimate and not recorded. The amniotic fluid volume remained normal in serial scans. At 34 weeks there was absent diastolic flow in the umbilical artery. The patient received corticosteroids and had a lower segment CS. A live baby girl with a birth weight of 1370g and Apgar score of 7/10 at 1 minute and 8/10 at 5 minutes was delivered. The acardiac twin weighed 3000g (Figure 1). The pump twin had respiratory distress syndrome for 3 days after delivery and was weaned off oxygen by day 4. She had no obvious congenital anomalies.

## Discussion

This is a case of a multiple gestation affected by the TRAP sequence which presented with a viable donor twin and non-viable acardiac twin at 26 weeks gestation and was managed conservatively until a CS was done at 34 weeks for foetal compromise with a resultant good outcome for the normal twin. Our patient booked for antenatal care in the second trimester and a routine ultrasound scan showed that the second twin was non-viable. Earlier diagnosis of TRAP is now possible with advances in first trimester ultrasound. In our case, doppler ultrasound of the acardiac foetus did not immediately pick up the reversed flow in the acardiac twin. Colour doppler would have been useful in tracing the vessels and showing the artery to artery anastomosis [6]. Due to limitation of resources and late presentation our management options were limited. We managed the patient with two weekly ultrasound scans and doppler velocimetry where we looked out for polyhydramnios and cardiac failure in the pump twin marked by absent diastolic flow in the umbilical artery. Other parameters like pulsatile blood flow in the umbilical vein and reversed blood flow in the ductus venosus would have aided in the diagnosis of cardiac failure in the pump twin [7]. Unfortunately, we were unable to measure the intrauterine size of the acardiac twin to allow us to estimate the relative size of the acardiac twin compared to the pump twin. The ratio of the weight of the acardiac twin to that of the pump twin was 0.46. Usually if the ratio of the weight of the acardiac to the pump twin is greater than 0.7, the risk of preterm birth is higher [8]. Other options for management not immediately available to us were laser, bipolar and radiofrequency coagulation of the umbilical cord as well as amnioreduction had the pregnancy been complicated by polyhydramnios. These procedures for in utero therapy are available at a small number of institutions in the developed world [7, 8]. That said, the best management of TRAP is not yet concluded and is controversial. Most cases of TRAP are diagnosed in the second trimester where it is impossible to predict the outcome. The pump twin may survive as in our case without any intervention in at least 50% of cases and so some authors recommend minimally invasive procedures if arrest of flow has not occurred by 16 weeks. There are studies that however report survival of 80 to 90% of pump twins in pregnancies that underwent in utero coagulation and ablation procedures [9].

## Conclusion

Pregnancies complicated by TRAP present a management challenge especially in low resource set up without advanced feto-maternal medicine units. Serial ultrasound scans and doppler velocimetry are useful in following up patients affected by TRAP sequence to assess the circulatory wellbeing of the pump foetus with the aim of deciding when to deliver.

## Competing interests

The authors declare no competing interests.
